# Experience Reverses the Red Effect among Chinese Stockbrokers

**DOI:** 10.1371/journal.pone.0089193

**Published:** 2014-02-24

**Authors:** Tengxiao Zhang, Buxin Han

**Affiliations:** Key Laboratory of Mental Health, Institute of Psychology, Chinese Academy of Sciences, Beijing, China; Monash University, Australia

## Abstract

Recent research has shown that the color red influences psychological functioning. Red is hypothesized to be linked to aggression and danger in evolution, and these links are enhanced by culture-specific uses of red. Thus, color meanings are thought to be grounded in biologically based proclivities and learned associations. However, to date, there has been no direct evidence for the influence of experience on the red effect. This study focused on whether experience could change the psychological effects of the color red. In the context of the Chinese stock market, contrary to the meaning generally associated with red as negative and green as positive, red represents a rise in stock price and green stands for a decrease. An experiment using a 2×2 between subjects factorial design demonstrated that red (compared with green) impaired Chinese college students’ performance on an IQ test (in accordance with the red effect), but the opposite effect was found among stockbrokers. These results provide direct evidence of learned color meanings, in support of the general model of color effect.

## Introduction

Color is one of the most common stimuli in human life, and its psychological effect has been widely investigated, with most studies focusing on the color red. Research on the effect of red in human competition demonstrated that competitors wearing red were more likely to win in Olympic games than were those wearing blue, because red is linked with aggression and dominance [1]. Subsequently, experimental studies confirmed this effect of the color red and provided explanations as to the mechanisms involved. Subtle red stimulus priming before IQ tests undermined intellectual performance in an achievement context [2]. Avoidance behavior (e.g., choosing easy items over difficult ones) and specific electrophysiological reactions were found as mediation variables [3]. Investigators inferred that the red effect was due to avoidance motivation induced by the color red [4]. Prior research has also shown that red induces avoidance motivational processes, such as worry. For example, exposure to the word “red” before IQ tests was related to greater feelings of worry and impaired performance in participants [5]. Physiological indicators of worry and anxiety, such as changes in heart rate variability, were also induced by the color red [6]. In summary, previous research shows that in achievement contexts, the color red undermines performance by inducing avoidance motivation and negative emotion.

Investigators hypothesized that avoidance motivation and negative emotion were induced by a link between red and feelings of aggression or danger. These psychological correlates of color are thought to be grounded in two sources, namely biologically based proclivities and learned associations. The links between color and meaning constitute a signal system created through a lengthy evolutionary process and strengthened by culture [7]. Studies of animal behavior provide evidence for the evolution hypothesis. Red in the appearance of some birds and primates is a natural symbol of aggression or social status; furthermore, animals tend to avoid those with appearances that are redder than their own [8], [9], [10]. It would be adaptive for humans if this system were inherited since it leads to the avoidance of conflicts with more powerful opponents. Meanwhile, the use of the color red in human societies might strengthen this biological signal system. In various cultures, red is commonly used to indicate danger to life, wrongful behavior, and failure. The red of stoplights and warning signs or the red ink used to correct errors are prototypical examples of the association between red and negative meanings [2]. These experiences are repeated throughout human life, thereby repeatedly reinforcing the association. Although many experiences support that the psychological meanings of the color red might be learned or enhanced during daily life, there is little empirical evidence for this hypothesis to date.

This study was designed to explore whether special experience with the color red could modify the normal psychological meanings associated with this color. Some professions involve daily experience with special color meanings. For example, red and green are used to indicate price changes in the stock market. Therefore, stockbrokers can be expected to show an increased sensitivity to red and green, due to their frequent experience with these colors and the associations of these colors to financial loss or gain. In the Chinese stock market, red stands for a rise in stock price and green indicates a decrease, which is the reverse of the typical use of red to denote negative meanings. Because of these special experiences, stockbrokers in China may have positive associations with the color red. As a consequence red might induce motivations of approach rather than of avoidance. Thus, we hypothesized that the color red has a positive effect on the psychological functioning of Chinese stockbrokers, because they connect red with money and success.

First, we aimed to confirm that the red effect reported in previous studies in Western societies is also found in a Chinese context. To this end, we tested whether a subtle red stimulus would impair performance on a subsequent IQ test among Chinese students. Next, we recruited a group of stockbrokers to explore whether they show a reversed red effect. We expected that red would enhance their intellectual performance due to their professional experiences concerning red and the resulting positive meaning of this color. We also measured negative emotion and achievement goals as process variables, exploring possible mediators between color and performance. Specifically, the tests of difficulty preference, achievement goals, visual matching, and color preference were employed to assess avoidance motivation of the participants.

## Methods

### Ethics Statement

All research reported here was approved by the Institutional Review Board of the Institute of Psychology, Chinese Academy of Sciences. Participants received modest monetary compensation for their participation, gave written informed consent, and were treated in accordance with the ethical standards expressed in the Declaration of Helsinki.

### Participants and Procedure

Because most stockbrokers in China are male, and in some studies the red effect was found only in male subjects [11], we only recruited male participants. A 2 (experience: college students or stockbrokers) ×2 (color: red or green) between subjects factorial design was used. Twenty-four college students and 24 stockbrokers were randomly assigned to one of two conditions (red vs. green) respectively. The stockbrokers had all worked in stock companies for at least one year, and their job was to buy and sell stocks for investors. The college students had no experience of working at a stock exchange. All participants had normal color vision.

The mean age of the college students was 23.46±2.83 years with a range of 20 to 31. Participants in the red group (n = 12) averaged 23.17±2.37 years of age, while those in the green group (n = 12) had an average age of 23.75±3.31 years. The number of years of education was 16.33±0.65 for the red group and 16.08±0.52 years for the green group. Neither age, *t*(22) = 0.50, *p = *.624, nor length of education, *t*(22) = 1.04, *p = *.308, significantly differed between the two groups.

The mean age of the stockbrokers was 24.79±1.84 years with a range of 22 to 29 years. The stockbrokers in the red group (n = 12) averaged 24.67±1.97 years of age and those in the green group (n = 12) averaged 24.92±1.78 years. The mean number of years of education was 15.58±0.52 in the red group and 15.58±0.52 in the green group. In addition, the mean duration of employment as brokers was 2.71±1.84 years for the red group and 2.33±0.96 years for the green group. There were no significant differences between the two groups in terms of age, *t*(22) = 0.33, *p* = .747, average length of education, *t*(22) = 0.00, *p* = 1.000, or mean employment duration, *t*(22) = 0.63, *p* = .538.

We integrated the procedures and measurements used in previous studies [2], [4], [5] into a computer program. Participants first completed the computerized tests, and afterwards they were asked to complete a paper-and-pencil questionnaire and a color vision test (to exclude cases of color blindness). The color vision test was administered at the end to avoid the possible priming effect of its colorful pictures. The questionnaire was used to assess participants’ awareness of the purpose of the experiment and whether they had seen the correct color during the tasks. Furthermore, it recorded their color preferences and demographic information. After the color vision test, participants were informed about the true purpose of the experiment and were offered the option of withdrawing their data from analysis.

At beginning of the computerized test, participants were informed that they were going to take an IQ test. They were asked to try their best to answer the questions on the test and were informed that their scores would be compared with that of other participants. These instructions were meant to create an achievement context. Then, three assessments were administered sequentially.

The first assessment measured various process variables including worry (“I worry that I may not do as well in the following IQ tests as I could”), difficulty preference (“I would like to take easier items,” indicating difficulty avoidance; and “I would like to take more difficult items,” indicating difficulty approach), and achievement goals (“My goal is to avoid performing worse than others on this task” which measured performance avoidance goals; and “My goal is to perform better than others on this task” which measured performance approach goals). All items were rated on a nine-point scale, from 1 (*not at all true of me*) to 9 (*very true of me*).

The second task measured avoidance motivation using a 12-item visual matching task. A target figure and two comparison figures were presented in each item. The participants had to choose which comparison figure was more similar to the target figure. One comparison figure had the same local elements as the target, and the other had the same global figure as the target. Greater similarity judgments based on local elements indicated greater avoidance motivation.

The third task was a subset of Raven’s Standard Progressive Matrices (Chinese version) [12]. Thirty items (those with odd serial numbers) were selected from the 60 items of the original test to reduce the duration of the experiment. The selection method ensured that the structure and difficulty of the subset was similar to the original test. This task was limited to six minutes.

Color manipulations were set before every task. The name of each task (the status, intuition, and reasoning tests, respectively) was first presented on a 19 cm×15 cm rectangle for five seconds on the screen before the task started. The task names were black and the rectangle was either red (R = 255, G = 0, B = 0) or green (R = 0, G = 255, B = 0).

All the participants completed the computerized tests on the same computer individually when they arrived at the lab. An assistant who did not know the purpose of the experiment started the program and left the participants alone. During the computerized tests, keyboard responses and reaction time were recorded.

## Results

Descriptive statistics, a 2×2 between subjects factorial design ANOVA, and chi-square tests were conducted for each relevant variable.

### Awareness of Color Manipulation and Purpose of Experiment

All participants correctly remembered the color they had seen during the experiment. No participants guessed that the experiment focused on color and intellectual performance, and not a single participant thought that color would affect their scores.

### Worry

No main effect was found for experience, *F*(1, 44) = 0.89, *p* = .352, or color, *F*(1, 44) = 1.46, *p* = .233, on the self-rating scale of worry. The interaction of experience and color was also not significant, *F*(1, 44) = 2.19, *p* = .146. Although the interaction was not significant, it is worth noting that for the college students, those in the red group reported greater worry (*M* = 3.83; *SD* = 1.59) than did those in the green group (*M* = 2.17; *SD* = 1.70), t(22) = 2.49, p = .021, Cohen’s d = 1.05.

### Avoidance Motivation

We found no main effects or interaction of experience and color in terms of difficulty preference, achievement goals, or visual matching.

Regarding color preferences, only one college student (4.2%) but eight stockbrokers (33.3%) reported that they liked red most. Conversely, twelve college students (50.0%) but no stockbroker (0.0%) reported that they disliked red most. Compared with college students, more stockbrokers liked red (χ^2^ = 6.70, *p*<.01, φ = 0.37), and most of them reported it to be a color they preferred in the context of their job. Similarly, more college students than stockbrokers disliked red (χ^2^ = 16.00, *p*<.01, φ = 0.58), and most students reported that red induced negative feelings such as nervousness and oppression.

### Intellectual Performance

The 30 items of the Chinese version of Raven’s Standard Progressive Matrices showed relatively high reliability, with a Cronbach’s alpha coefficient of 0.785 and a split-half reliability of 0.792.

**Figure 1:**
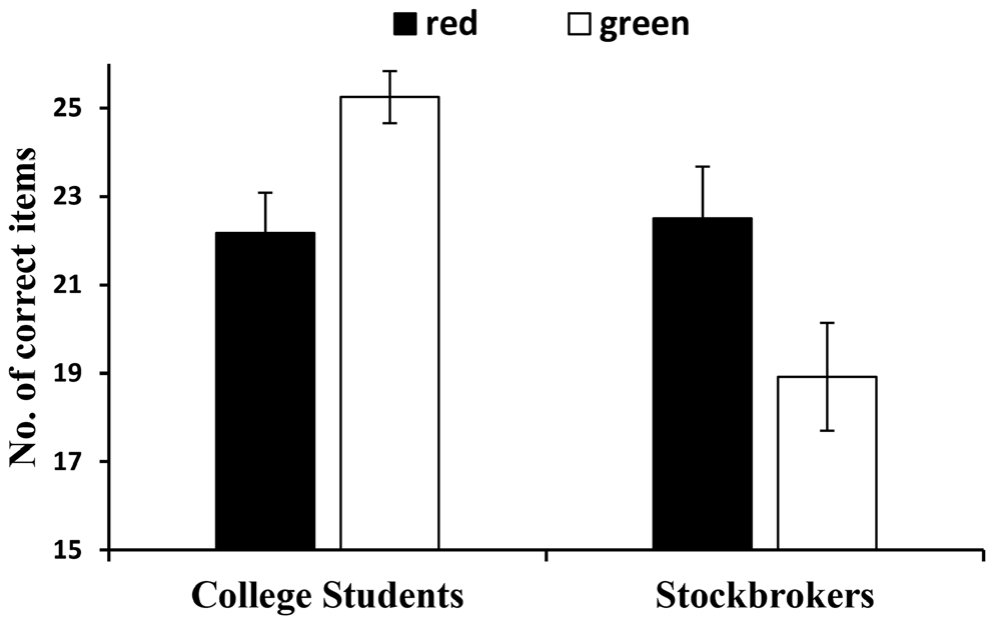
The effect of color on performance on Raven’s Standard Progressive Matrices The college students in the red group (n = 12) performed worse than did those in the green group (n = 12). Conversely, the stockbrokers in the red group (n = 12) performed better than did those in the green group (n = 12). Error bars indicate standard error of test scores.

There was a significant main effect of experience, *F*(1, 44) = 8.84, *p* = .005, partial eta^2^ = .17, indicating that college students scored higher on the intellectual test (*M = *23.71; *SD* = 3.06) than did stockbrokers (*M* = 20.71; *SD* = 4.46). The main effect of color was not significant, *F*(1, 44) = 0.06, *p* = .806. More importantly, as predicted, a significant interaction of experience and color was found, *F*(1, 44) = 10.91, *p* = .002, partial eta^2^ = .20. As Figure 1 shows, the interaction supported the hypothesis, as college students in the red group (*M* = 22.17; *SD* = 3.19) performed worse than those in the green group (*M* = 25.25; *SD* = 2.05), but stockbrokers showed the reverse pattern, with participants in the red group (*M* = 22.50; *SD* = 4.08) performing better than those in the green group (*M* = 18.92; *SD* = 4.23). Ancillary analyses indicated that the red versus green contrast was significant both for college students, *t*(22) = 2.82, *p* = .010, Cohen’s *d = *1.20, and stockbrokers, *t*(22) = 2.11, *p* = .046, Cohen’s *d = *0.90.

## Discussion

The interaction of experience and color confirmed our hypothesis that the red effect would reverse among stockbrokers. The results of the college students were consistent with those of previous studies [2], [5], confirming the existence of the red effect among Chinese participants. Compared with the color green, the subtle red stimulus before the IQ test undermined test performance among college students. However, among stockbrokers, red enhanced the performance on the IQ test. The effect was implicit, as participants were not aware of the influence of color.

This reversed red effect is probably due to the reversed professional color–meaning links among stockbrokers. Red and green are used frequently in the context of the stock market and as a result, they might be easily linked with positive (i.e., financial gain and success) and negative (i.e., financial loss and failure) psychological meanings, respectively. Due to their professional experience, stockbrokers were more likely to approach red and avoid green.

Participants’ color preference provides further support for this interpretation. College students tended to report they did not like red because it aroused negative feelings. Subjects’ avoidance of the red threat stimulus is in accordance with the tenets of evolutionary psychology. However, the stockbrokers tended to report liking red better because in their work experience, red stood for a rise in stock price. This implies that experience may change the psychological meanings associated with colors.

Elliot and Maier [7] proposed six basic premises for the general model of color and psychological functioning. Our study provides new evidence in support of this model. The effect of color was established, supporting the idea that color carries psychological meaning. Furthermore, the psychological processes evoked by color affect the ensuing motivated behavior. Even the mere perception of color can exert unconscious influence on psychological functioning. Most importantly, we found direct evidence for learned associations between color and psychological meaning. Repeated pairings of colors with experience can contribute to a new color effect, and even reverse the typical effect, despite its roots in evolution and cultural associations.

Previous research has shown the visual matching task to be an effective method of measuring avoidance motivation; indeed, it was found that priming with the color red induced more similarity judgments based on local as opposed to global elements, indicating increased avoidance motivation. However, in this study, the color red did not significantly affect the participants’ judgments based on local elements. This unexpected result might be due to a culture-specific response strategy among Chinese participants. Although the instructions emphasized that judgment should be based on participants’ intuition for each item, participants still tended to follow a rule in their decisions throughout the test. For example, if a participant made his judgment based on local elements on the first item, he would tend to use the same rule in subsequent judgments. Thus, the scores on the test were likely to be zero or full marks. This indicates that, the test was perhaps not measuring what it is supposed to measure.

A further limitation of our study is the use of the RGB method for manipulating color. A better approach would be to use a spectrophotometer to control the lightness and chroma of the color stimuli [13]. Furthermore, the sample sizes in our study are relatively small, although in line with other studies of this type. These limitations should be addressed by future studies.

As for future research, further evidence is needed to confirm that the color effect is part of a biological signal system. Except for research on animal behavior [9], there is little direct evidence in support of the notion that the red effect originates in biologically based proclivities. If the red effect was present among neonates or was stronger in more evolutionarily basic contexts, this would constitute new evidence for its biological origins.

Finally, our research was conducted in an achievement context. Context has been proven to be a moderating factor in studies of color effect [14]. Under the relational context, red may enhance the perception of attractiveness between sexes [15], [16]. Furthermore, red clothes may predict women’s attitudes toward sexual behavior [17] and red lipstick may increase the amount of tips waitresses receive [18]. Researchers agree that red is linked to love and sex in a relational context, and as a result it could induce a different psychological effect than in an achievement context [19], [20]. Thus, whether experience can modify color effects in other contexts is also a question to be answered by future research.
